# Effect of Basalt Fiber Diameter on the Properties of Asphalt Mastic and Asphalt Mixture

**DOI:** 10.3390/ma16206711

**Published:** 2023-10-16

**Authors:** Bo Li, Minghao Liu, Aihong Kang, Yao Zhang, Zhetao Zheng

**Affiliations:** 1 College of Civil Science and Engineering, Yangzhou University, Yangzhou 225100, China; libo@yzu.edu.cn (B.L.); lmhanksuper@163.com (M.L.); yaozhang@yzu.edu.cn (Y.Z.); zzt451873798@163.com (Z.Z.); 2 Research Center for Basalt Fiber Composite Construction Materials, Yangzhou 225127, China

**Keywords:** basalt fibers, diameter, asphalt mastic, monofilament pullout test, asphalt mixture

## Abstract

In this study, basalt fiber having two types of diameters (16 μm and 25 μm) was selected and added to asphalt mastic and asphalt mixtures using different fiber proportions. The influences of fiber diameters and proportions on the properties of asphalt mastic and mixtures were studied. The adhesion behavior of the fiber-asphalt mastic (FAM) interface was evaluated by a monofilament pullout test, and the rheological properties of FAM were evaluated by temperature sweep, linear amplitude sweep, and bending beam rheological tests. In addition, the high-temperature stability, intermediate and low-temperature cracking resistance, and water stability of fiber-modified mixtures were studied by wheel tracking, ideal cracking, a low-temperature bending beam, and a water-immersed Marshall test. The results showed that the interface adhesion behavior between 16 μm fiber and asphalt mastic was more likely in the fiber failure mode at both −12 °C and 25 °C. Adding basalt fiber can significantly improve the high-temperature and fatigue properties of asphalt mastics. Moreover, 16 μm fiber had a better modifying effect on asphalt mastic than 25 μm fiber. The same enhancement trend can be observed in asphalt mixtures. Basalt fibers with 16 μm diameters can improve the high-temperature performance of asphalt mixtures more significantly. In addition, 16 μm fiber could sharply enhance the cracking performance of the mixtures at intermediate and low temperatures, while the enhancing effect of 25 μm fiber on the mixture is insignificant, though both diameters of the fibers have a minor effect on the water stability.

## 1. Introduction

The traditional asphalt mixture is a composite material that is mainly obtained by mixing aggregates, asphalt, and fillers. Due to its excellent performance, it is widely used in different types of road engineering fields [1,2,3]. The service function of asphalt pavement has drastically deteriorated due to the increasing traffic volume and the effects of climate change. And the pavement structure suffers distress such as cracking, aging, rutting, water seepage, etc. [1]. Therefore, searching for a more durable, safe, and environmentally friendly pavement material has become a research hotspot in global transportation construction [4,5,6,7], such as incorporating fibers and anti-rutting agents.

Since the 1990s, lignin fibers have been added to Stone Mastic Asphalt to increase its stability [8]. Due to their fluffy structure and rough surface, lignin fibers can better adsorb free asphalt and increase the stability of the asphalt mixture [9]. Other types of fibers, such as glass, polyester, and polyacrylonitrile fibers, are also frequently employed in asphalt mixtures and can vary in their ability to enhance the overall performance of asphalt mixtures [10,11,12]. As an environmentally friendly material [13], basalt fiber has been widely studied as an additive because of its unique advantages of high-temperature resistance, high strength, and stable chemical properties [11]. Two levels—asphalt mastic and asphalt mixture—can be used to examine the modification impact of basalt fiber on asphalt materials. Although asphalt mastic has a smaller proportion in the mixture compared with that of aggregates, its role in the asphalt mixture is irreplaceable. The mastic’s composition, microstructure, and rheological properties directly affect the performance of asphalt mixtures [14,15,16,17]. Using fiber materials is an effective way to improve the properties of asphalt mastic, and fiber types, characteristics, and content also have other enhancement effects on asphalt mastic [18,19]. Xing et al. observed the impact of basalt fibers on the asphalt material’s performance. The results indicated that basalt fibers can improve the toughness of asphalt [20]. Qin et al. investigated the enhancing effect of fiber types on asphalt mastic properties. They found that basalt fiber presented the best overall performance, and basalt fiber with a length of 6 mm had the best-enhancing effect on the cracking resistance [21].

Studies have shown that basalt fibers can significantly enhance the asphalt mixture’s performance. Ramesh et al. determined that the RAP material of the AC mixture accounted for 30% by weight of the total aggregate and the chopped basalt fiber accounted for 6% by weight of the binder, which met the target AC mixture cracking standard, using the standard semicircular bending (SCB) test. [22]. Song et al. combined an indoor SCB test and digital image correlation method to analyze the fracture characteristics of DTC phase-change asphalt mix reinforced by basalt fiber. The research results revealed that basalt fiber improved the fracture toughness of SMA and increased its resistance to low-temperature cracking [23]. Lou et al. investigated the reinforcing ability of basalt fibers of various lengths on asphalt mixtures and found that mixed-length basalt fibers further improved mix crack resistance [24]. Pirmohammad et al. analyzed the impacts of the content and length of basalt fibers on the fracture toughness of the mixture through SCB experiments. They found that 0.3% basalt fiber with a length of 4 mm had the best-improving effect on fracture toughness [25]. Zhang et al. used the Hamburg rutting experiment to test an OGFC asphalt mixture with different contents of basalt fiber. They found that 0.15% basalt fiber had the best effect on mixture performance [26]. Lou et al. used uniaxial penetration tests to find that the permeability of the ultra-thin wearing course (UTWC) reached the maximum value of AC-10 when the fiber content of 6 mm fiber was 0.3% [27]. Luo et al. discussed how fiber type and dose affected the mixture’s high-temperature stability at the Micro-surfacing, showing that the impact of fiber on the enhancement of rutting resistance at the Micro-surfacing was relatively small [28]. Lou et al. explored the impact of fiber length on various gradation mixtures. They found it significantly affected the hot mix asphalt’s fatigue, intermediate, and low-temperature cracking resistance. In addition, they had no discernible impact on water stability or high-temperature deformation performance [29]. The asphalt mixture’s performance at low temperatures first increased and then decreased as the amount of basalt fiber in the asphalt mixture increased. The results revealed that the asphalt mixture performance at low temperatures was best at the particular amount of basalt fiber [30]. This fiber agglomeration phenomenon in the mixture will have detrimental effects. To increase the mixture’s water stability, the optimal amount and length of fibers can create a stable three-dimensional network structure [28]. This is primarily due to the fibers’ even distribution throughout the asphalt mixture, which creates a three-dimensional network structure. It can decrease the fluidity of asphalt close to the fiber and then enhance the performance of asphalt mastic and asphalt mixture, resulting in prolonging the pavement’s service life.

In summary, fiber’s properties are the primary indexes causing the reinforcing effect on the asphalt mixture. The diameter of basalt fiber directly affects the amount of fiber monofilaments and the specific surface area of fiber at a given weight content. Hence, its influence on the mixture is also crucial. There are, however, few investigations on the enhancing ability of fiber diameter on the characteristics of asphalt mixtures and even fewer on various grades of asphalt mixtures. Due to the limitations of the production process, the most widely used diameters of basalt fiber are 7 μm, 16 μm, and 25 μm. The price of basalt fiber with a diameter of 7 μm is much higher than the other two, and it is mainly used in the textile industry. Meanwhile, basalt fiber with diameters of 16 μm and 25 μm is mainly used in road engineering. In addition, there might be the phenomenon of mixed diameters in the same batch of fibers produced by the same manufacturer. Subsequently, the suitability of using this mixed fiber type in engineering must be further explored. As the research objects, two distinct gradations (AC-13 and AC-20) and two different diameters of basalt fibers (16 μm and 25 μm) were chosen. And multiple experiments were conducted to investigate the impact of basalt fibers on the properties of fiber-asphalt mastic and fiber-asphalt mixtures. The findings can provide guidelines for future studies and technical applications of basalt fibers in asphalt mixtures.

## 2. Materials and Methods

### 2.1. Materials

#### 2.1.1. Asphalt

Jiangsu Nantong Tongsha Asphalt Technology Co., Ltd. (Nantong, China) supplied us with SBS-modified asphalt (PG76-22), and its properties are listed in Table 1.

#### 2.1.2. Fiber

**Figure 1:**
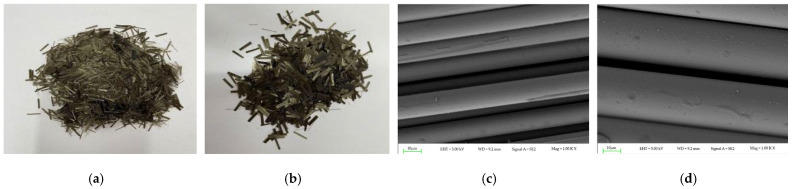
Macroscopic and microscopic diagrams of basalt fiber morphology; (**a**) 16 μm Basalt fiber; (**b**) 25 μm Basalt fiber; (**c**) Microscopic diagrams of 16 μm basalt fiber; (**d**) Microscopic diagrams of 25 μm basalt fiber.

The basalt fibers used in this research are short-chopped and have two different diameters (16 μm and 25 μm). Jiangsu Tianlong Basalt Continuous Fiber Co., Ltd. (Yangzhou, China). supplied the fibers. Basalt fiber is solid, can resist corrosion and high temperatures, and has excellent performance indicators (shown in Table 2). Figure 1 displays the macro-micro diagram of the basalt fibers used.

#### 2.1.3. Filler

The mastic in the experiment is made by SBS asphalt and the mineral powder filler using a weight ratio of 1:1 [32], and the mineral powder in the experiment is selected from Zhenjiang high-capital limestone ore powder; the property indices of the mineral powder filler are shown in Table 3.

#### 2.1.4. Aggregate and Gradation

Limestone and basalt aggregates were used in this investigation. Table 4 displays the findings for determining apparent density and gross bulk density. The fiber-reinforced asphalt mixture is graded as AC-13 and AC-20, and the data for this gradation is displayed in Table 5 and Table 6.

### 2.2. Specimen Preparation

Before the experiment, SBS-modified asphalt, mineral powder, and basalt fiber were heated in the oven at 175 °C for 2 h to make the asphalt flow and to remove the water in the mineral powder and fiber. Based on the previous research, the fiber content in asphalt mastic and asphalt mixture was 5% and 0.3% [32], respectively.

First, the weight ratio of mineral powder and asphalt was 1.0. Then, it was mixed with asphalt mastic at 600 r/min until the mineral powder was evenly distributed in the asphalt mastic. Finally, 5% fiber to the total weight of asphalt mastic was added to the material three times, and it was stirred with a stirring bar until the fiber was completely dispersed in the asphalt.

As to the preparation of asphalt mixture samples, aggregate, fiber, mineral powder, and asphalt were heated at 175 °C. The aggregate was then mixed with fiber and stirred for 90 s until the fiber was evenly dispersed between the aggregates. Then, asphalt and aggregates were stirred evenly, and finally, the mineral powder was added for stirring to obtain the fiber asphalt mixture. Six sets of samples were fabricated: samples without doped fibers (blank); only 16 μm basalt fiber specimens (16 μm); only 25 μm basalt fiber specimens (25 μm); and samples with mass ratios of 16 μm to 25 μm basalt fiber of 1:1 (1:1), 1:2 (1:2), and 2:1 (2:1), respectively.

### 2.3. Test Methods

**Figure 2:**
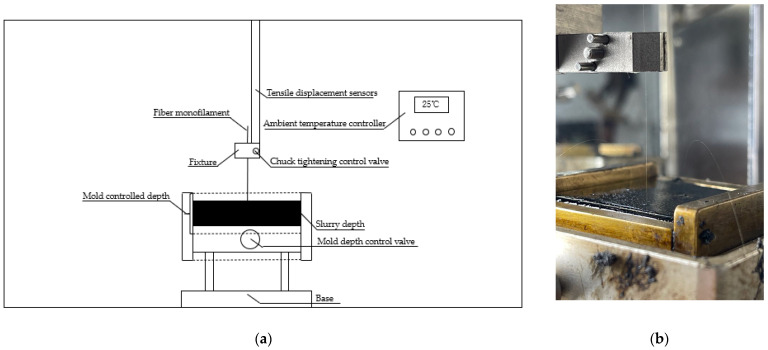
Fiber pullout test: (**a**) Schematic diagram of a monofilament-asphalt pullout device; (**b**) Actual test diagram.

In this study, the fiber-asphalt interface adhesion behavior was evaluated using the monofilament fiber-asphalt pullout instrument, which was self-developed. The experimental process is shown in Figure 2. As there is presently no approach for investigating asphalt mastic, the experimental standards for the fiber asphalt mastic in this study were the same as the asphalt experimental standards. Fiber asphalt mastic was tested with DSR and BBR instruments, while UTM-25 was used to test fiber asphalt mixtures.

#### 2.3.1. Monofilament Fiber Pullout Test

**Figure 3:**
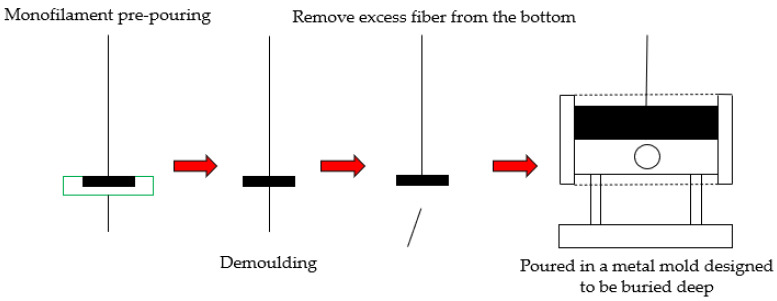
Schematic diagram of monofilament fiber-asphalt pullout specimen production.

In this study, the monofilament fiber-asphalt pullout instrument was used. The following were the preparation steps: (1) the monofilament fiber was first peeled off with metal tweezers; (2) the monofilament was passed through the soft rubber mold with a diameter of 2 cm in the round groove, and then the asphalt mastic was poured; (3) the excessive fiber monofilament at the bottom was removed after cooling; (4) the specimen was moved to the wire pullout die and poured to the required depth for the experiment, as shown in Figure 3; After the specimen was placed in the instrument for 2 h, the pull-out test was carried out, and the loading rate was set to be 1 mm/s. The maximum tensile load and fiber tensile displacement curves at −12 °C and 25 °C were tested. The calculation formulas are shown in Equations (1)–(3).

(1) S=π×d×L

(2) $$\tau_{m}=\frac{F_{\max}}{S}$$

(3) $$W_{a}=\int_{0}^{x_{a}}Fd(x)$$

Here *F* is the pulling force in the pulling period, cN; *S* is the cohesion area between asphalt and fiber, mm^2^; *d* is the diameter of the fiber monofilament, mm; *x* is the real-time displacement during the pullout process, mm; $x_{a}$ is the displacement corresponding to the maximum pulling force, mm; *L* is the fiber embedding depth, mm; $W_{a}$ is the energy generated before the fiber-asphalt interface begins to debond, which is the integral of the tensile force and the displacement of the fiber before the moment of complete debonding, 10^−2^∙mJ.

#### 2.3.2. Temperature Sweep Test of Asphalt Mastic

**Figure 4:**
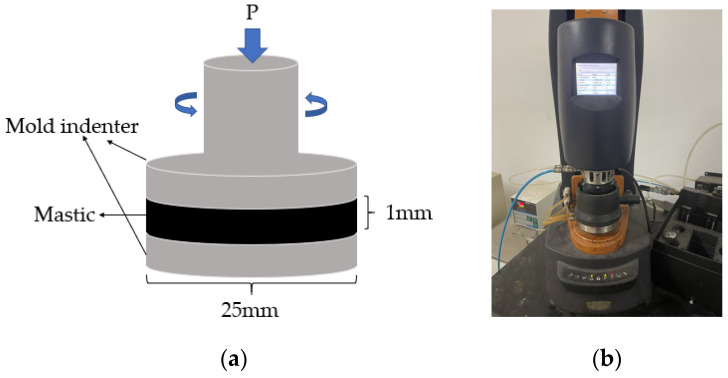
Temperature sweep test: (**a**) experimental model; (**b**) actual test diagram.

The DSR temperature sweep test was conducted using the dynamic shear rheometer from TA Instruments according to the AASHTO T 315-12 specification [34], as shown in Figure 4 with the temperature set to 52 °C~82 °C. The fiber asphalt mastic was injected into an elastic rubber mold with a diameter of 25 mm and a thickness of 2 mm. After the sample was cooled, it was removed and set on the fixture, the excess material was tripped off, and the test data were collected at intervals of every 6 °C. The final data were the average value of three duplicates.

#### 2.3.3. Linear Amplitude Sweep Test of Asphalt Mastic

**Figure 5:**
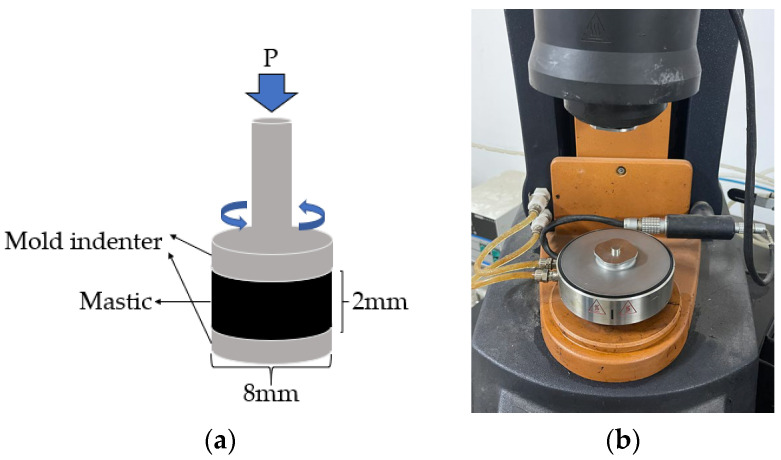
Linear amplitude sweep test: (**a**) experimental model; (**b**) mold indenter.

The fatigue performance of asphalt was assessed using the linear amplitude sweep (LAS) technique following the AASHTO TP 101-12 guidelines [35]. The test uses three strain levels of 2.5%, 5.0%, and 10% at a temperature of 25 °C as shown in Figure 5. The diameter of the mold indenter is 8 mm, and the gap is 2 mm. The fatigue life *N_f_* was calculated in Equation (5), and the coefficients A_35_ and B were calculated by Equations (6) and (7). The final data were averaged across three samples.

(4) $$G^{\prime }(\omega )=\left|G^{*}\right|(\omega )\times \cos\delta (\omega )$$

(5) $$N_{f}={\left(\gamma_{\max}\right)}^{-B}$$

(6) $$A_{35}=\frac{f{\left(D_{f}\right)}^{k}}{k{\left(\pi I_{D}C_{1}C_{2}\right)}^{\alpha }}$$

(7) B=2α

where $\gamma_{max}$ is the maximum strain based on the applied load; *f* is the frequency of loading, which is 10 Hz; *k* is the coefficient and the formula is *k* = 1 + (1 − *C*_2_)·*α*; *C*_1_, *C*_2_, *D_f_*, *α* is calculated rheological parameter; *I_D_* is the original value of |G*| from the 1.0 percent applied strain interval, (MPa); |G*| is the dynamic modulus in the test, (MPa); *δ*(*ω*) is the phase angle of each frequency, (°); *G*′(*ω*) is the converted storage modulus.

#### 2.3.4. Bending Beam Rheometer Test of Asphalt Mastic

**Figure 6:**
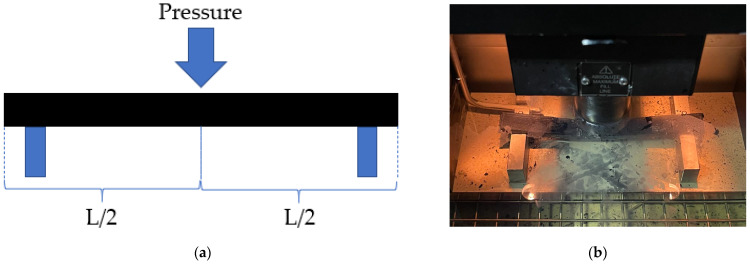
BBR test: (**a**) BBR experimental model; (**b**) actual test diagram.

The low-temperature characteristics of fiber-modified asphalt were investigated using a TE-BBR-F type bending beam rheometer according to the AASHTO TP 125-16 specification [36]. The test plot is shown in Figure 6. The test temperatures are from −6 °C to −24 °C, setting 6 °C as the interval. The sample was poured into a stainless-steel mold of 125 × 12.5 × 6.25 mm, and a water bath in ethanol was placed to maintain the experimental temperature before testing. The creep stiffness (S) and creep rate (m) of the beam sample were used to assess the fiber mastic’s low-temperature performance. Formulas (8)–(10) display the calculation mechanism for m and S.

(8) $$S(t)=\frac{1}{D}=\frac{\sigma }{\varepsilon }=\frac{Pl^{3}}{4bh^{3}\delta (t)}$$

(9) $$\log S^{\prime }(t)=A+B\left[\log(t)\right]+C{\left[\log(t)\right]}^{2}$$

(10) $$\left|m(t)\right|=\frac{d\left[\log S^{\prime }(t)\right]}{d\left[\log(t)\right]}=B+2C\left[\log(t)\right]$$

where *S*(*t*) is the real-time creep stiffness of the beam at 60 s (MPa); *m*(*t*) is the real-time creep rate; *P* is a constant load of 100 g; *L* is the length of the asphalt mastic beam, (mm); *b* is the sample width of the asphalt mastic beam, (mm); *h* is the sample height of the asphalt mastic beam, (mm); *δ*(*t*) is the real-time deflection of the specimen at 60 s, *S*′(*t*) is an estimate of the creep stiffness of flexural over time (MPa), and *A*, *B*, and *C* are the regression coefficients.

#### 2.3.5. High-Temperature Stability Test of the Mixture

**Figure 7:**
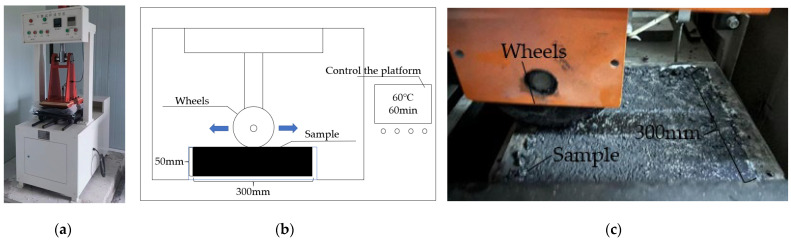
Rutting test: (**a**) QCX-4 Rut plate former; (**b**) Rutting experimental model; (**c**) Actual test diagram.

The mixture was formed into a cube specimen with dimensions of 300 × 300 × 50 mm by the rutting plate roller described in detail in JTG E20-2011 [31]. After the specimen was formed, it was first put into the experimental cabin for heat preservation, and the experiment was carried out after the specimen reached 60 °C, as shown in Figure 7. The rutting resistance of asphalt mixtures is assessed using the dynamic stability index, calculated by Equation (11).

(11) $$DS=\frac{(t_{2}-t_{1})\times N}{d_{2}-d_{1}}\times C_{1}\times C_{2}$$

where DS is the dynamic stability of the asphalt mixture (times/mm); *t*_1_ and *t*_2_ are the times of the experiment, usually 45 min and 60 min; *d*_1_ and *d*_2_ are the related deformations of *t*_1_ and *t*_2_ (mm); *C*_1_ is the correction coefficient of the experimental instrument, and it is set as 1; *C*_2_ is the correction coefficient of the specimen, and it is set as 1; *N* is the rolling number of wheels per minute, which is 42 times/min.

#### 2.3.6. IDEAL-CT Test of Mixture

**Figure 8:**
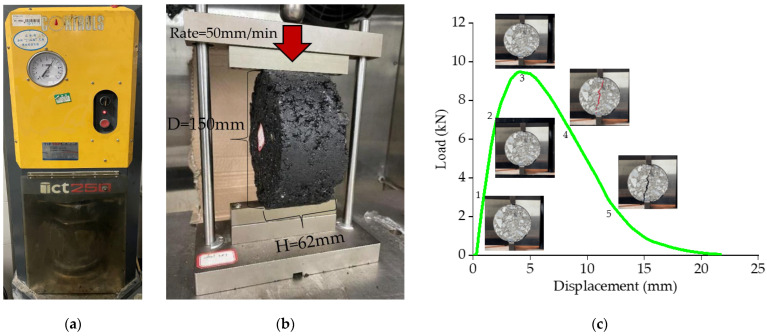
IDEAL-CT test: (**a**) Ict-Rotary compactor; (**b**) Actual test diagram; (**c**) IDEAL-CT test load-displacement curve.

The intermediate-temperature cracking resistance of fiber asphalt mixtures was studied using the “ideal cracking test” (IDEAL-CT). The 150 mm diameter and 62 mm height specimens were fabricated by the Ict-Rotary compactor. The specimens and the indenter were insulated to 25 °C and tested, as shown in Figure 8c. The *CT_index_* was obtained, and the crack initiation energy *G_r_* (the work done in the area corresponding to points 0–3 in Figure 8b) and the total fracture energy *G_f_* (the work done in the region corresponding to points 0-endpoint in Figure 8b over the cracking area) were adopted to assess the anti-crack ability at intermediate temperature. Equations (12) and (13) display the indicator computation.

(12) $$CT_{index}=\frac{G_{f}}{\left|m_{75}\right|}\times \frac{l_{75}}{D}$$

(13) $$\left|m_{75}\right|=\left|\frac{\left(p_{85}-p_{65}\right)}{\left(l_{85}-l_{65}\right)}\right|$$

where *CT_index_* is the index of the cracking test; $\left|m_{75}\right|$ is the absolute value of the gradient of 75% of the post-peak section; $G_{f}$ is the energy of fracture, (J/m^2^); $l_{75}$ is the displacement of 75% of the post-peak section, (mm); *D* is the diameter of the specimen, (mm).

#### 2.3.7. Low-Temperature Bending Beam Test of the Mixture

**Figure 9:**
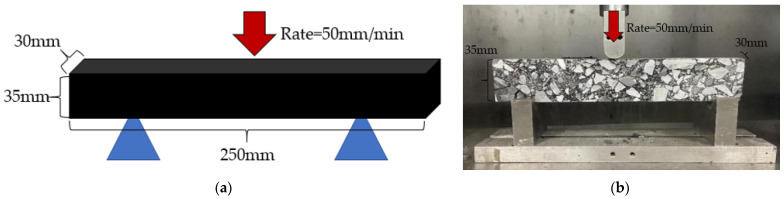
Bending beam test: (**a**) Model of test; (**b**) Actual test diagram.

The fiber asphalt mixture was made into a 30 × 35 × 250 mm prismatic beam specimen and tested after 2 h of insulation at −10 °C, as shown in Figure 9. Then, it was determined what the bending stiffness modulus *S_B_*, maximum bending strain $\varepsilon_{B}$, and bending tensile strength *R_B_* were. Equations (14)–(16) display the calculating formula.

(14) $$R_{B}=\frac{3LP_{B}}{2bh^{2}}$$

(15) $$\varepsilon_{B}=\frac{6hd}{L^{2}}$$

(16) $$S_{B}=\frac{R_{B}}{\varepsilon_{B}}$$

where *b* is the actual width of the beam, (mm); *h* is the sample height of the beam, (mm); *L* is the length of the beam, (mm); *P_B_* is the maximum load (N); *d* is the mid-span deflection (mm).

#### 2.3.8. Water Stability Test of the Mixture

**Figure 10:**
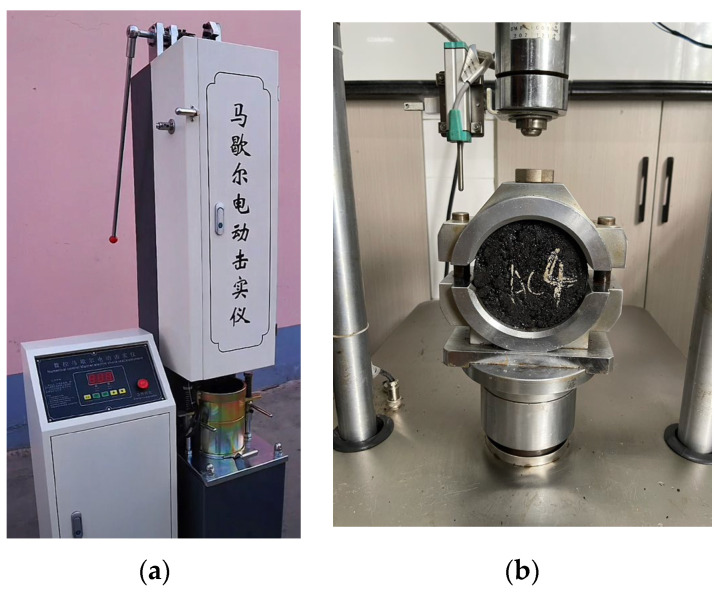
Water stability test: (**a**) Automatic Marshall compactor; (**b**) Schematic for water stability test.

Cylindrical asphalt mixture specimens fabricated by gyratory compaction were closer to those compacted in the field by a roller compactor [37]. Since the water-immersion Marshall test was conducted according to the specification of JTG E20-2011, the cylindrical specimen with a diameter of 101.6 mm and height of 63.5 mm was fabricated by the Marshall compactor, as shown in Figure 10. The specimens for the water immersion Marshall test were divided into two batches and immersed entirely in water at 60 °C; one batch was removed for the stability test after two hours, while the other batch was removed and tested after 48 h. The stability was expressed by Equation (17).

(17) $$MS_{0}=\frac{MS_{1}}{MS}\times 100$$

where *MS*_0_ is the residual stability of the specimen, (%); *MS*_1_ is the Marshall stability of the specimen after 48 h of water bath, (kN); *MS* is the Marshall stability after 2 h of water bathing (kN).

## 3. Results and Discussion

### 3.1. Fiber-Asphalt Interface Adhesion Characteristics

**Figure 11:**
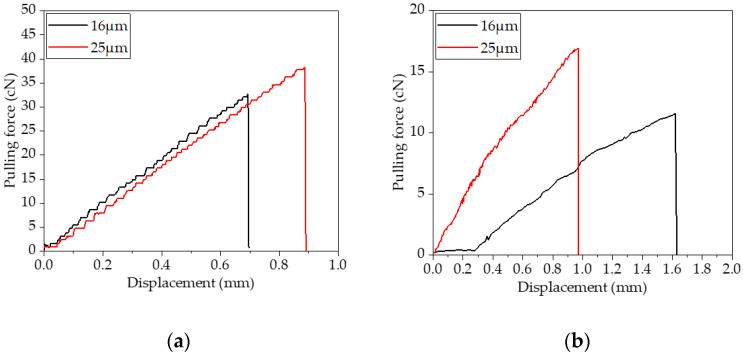
Fiber-SBS-modified asphalt mastic monofilament pullout curve: (**a**) −12 °C; (**b**) 25 °C.

**Figure 12:**
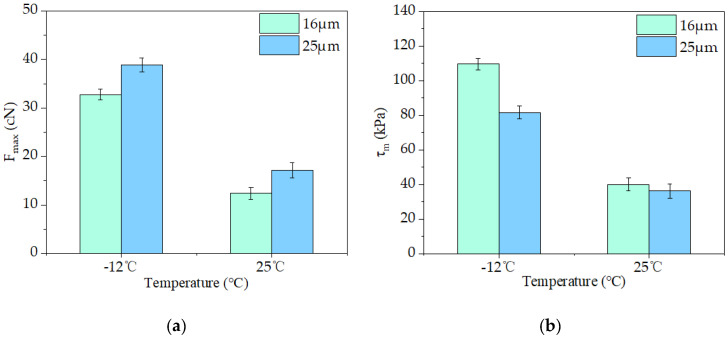
Fiber-asphalt interface adhesion parameters (the error bars illustrate the standard deviations of testing results): (**a**) *F*_max_; (**b**) *τ_m_*.

This experiment aims to analyze the bonding characteristics between fiber monofilaments and asphalt mastic at −12 °C and 25 °C. The pulling force-displacement curves are shown in Figure 11 and Figure 12.

Figure 11a demonstrates that, at −12 °C, the pulling force of the two fibers increases roughly linearly as displacement increases. When the pulling force reached a certain level, it dropped sharply to 0, and the fiber was broken. This shows that at −12 °C, the failure is more likely in fiber fracture mode. Experiments showed that the cohesion ability between asphalt mastic and fiber was very strong at low temperatures. Since the asphalt mastic hardened at lower temperatures and there was almost no viscous flow state, the pulling force mainly acted on the fibers, so the fibers broke. In contrast, the failure displacement of 25 μm basalt fibers was about 0.9 mm, which was greater than 0.7 mm for the 16 μm basalt fibers. This demonstrated that the maximum tensile force and elongation at the breaking point the fiber could withstand at low temperatures were slightly higher than those of 16 μm basalt fiber because 25 μm basalt fiber had a slightly larger diameter than 16 μm basalt fiber.

At 25 °C, the tensile force of two fibers with different diameters gradually increased with the increase in tensile displacement in the stretching process. The interface damage between them and asphalt mastic eventually displayed a violent fracture, showing that the fiber fracture mode was still present. As shown in Figure 11b, the displacement of the 16 μm basalt fiber when it was pulled off was close to 1.6 mm at the breaking point, which was greater than 0.97 mm of the 25 μm fiber. Because at 25 °C, asphalt mastic presented a viscoelastic state, the restraining effect on the fiber was smaller than that at −12 °C. Due to the large contact area between basalt fiber monofilament with a diameter of 25 μm and asphalt mastic, the adhesion area between asphalt mastic and the fiber was large [11]. Therefore, more pulling force was needed to make the same displacement; basalt fiber with a diameter of 25 μm had a much higher maximum pulling force than basalt fiber with a diameter of 16 μm.

Since the interface failure of two different diameters of fibers and asphalt mastic was manifested as fiber fracture mode at both −12 °C and 25 °C, there is no interface debonding work (*W_a_* was 0).

Figure 12a shows that the pulling force *F*_max_ of both fibers was higher at −12 °C than at 25 °C, and the fiber’s fracture strength parameters were in line with its size. The pulling force of 25 μm fiber in asphalt mastic was larger than that of 16 μm fiber at the same temperature. Figure 12b demonstrates that, nevertheless, the adhesion strength between fiber and asphalt mastic increases with the decreasing diameter of the fiber. At −12 °C, the interfacial adhesion strength of 16 μm basalt fibers increased by 34.18% compared to 25 μm fibers, and at 25 °C, this value increased only by 11.01%. This was due to the different diameters of fiber, and its cohesion area with the asphalt was also different. From Equation (2), it can be seen that in the case of *F*_max_, it was not much different; the larger the *S*, the smaller the interface adhesion strength.

### 3.2. High-Temperature Performance Analysis of Fiber Asphalt Mastic

**Figure 13:**
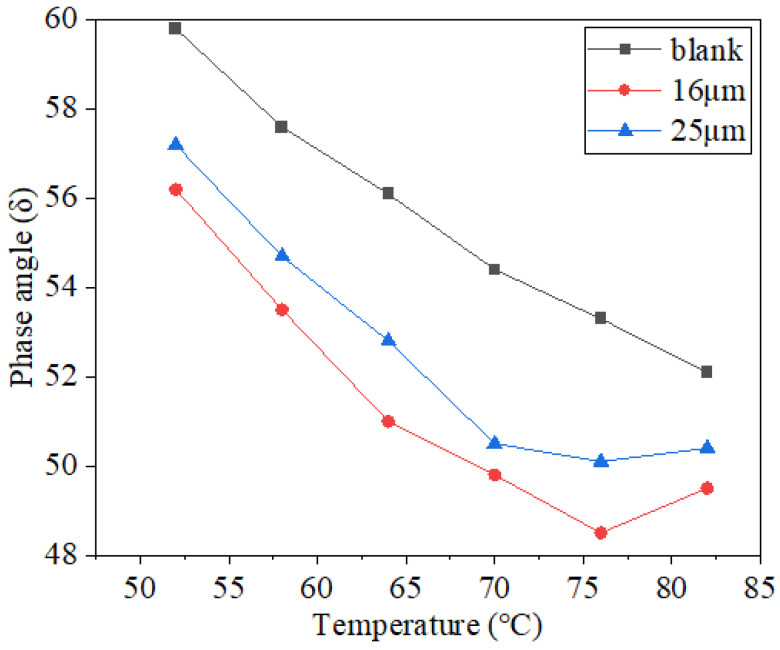
Test results for phase angles.

**Figure 14:**
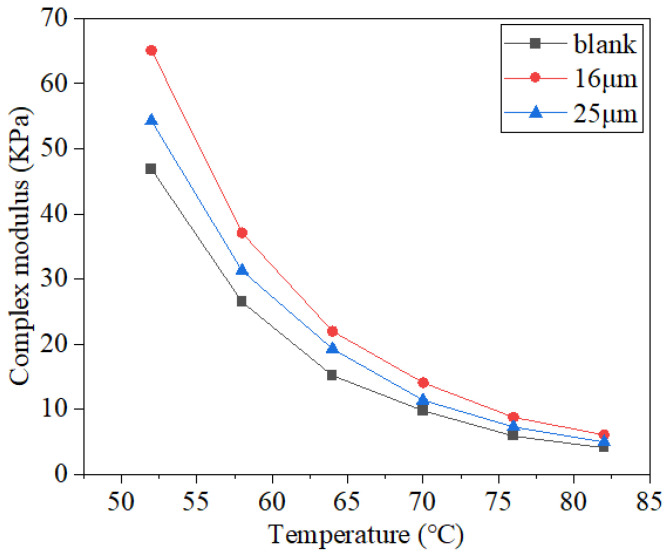
Test results for complex modulus.

**Figure 15:**
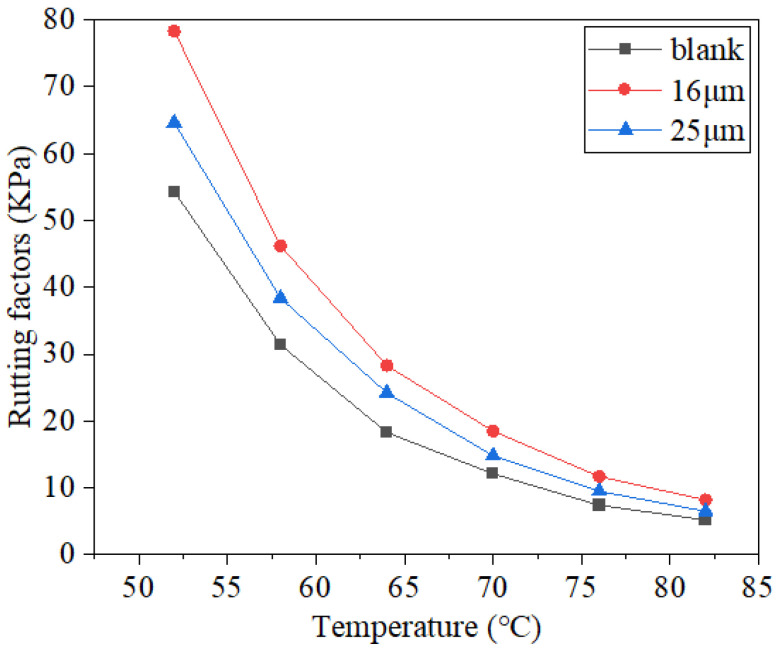
Test results of the rutting factor.

The relative amount of elastic deformation in asphalt could be characterized using the phase angle index, which increases as the phase angle decreases and makes the deformation caused by the load easier to recover. The complex modulus could be used to express the deformation resistance of a material, which is the ratio of the shear stress and strain. The rutting factor is usually used to characterize the rutting resistance of asphalt. The test results are illustrated in Figure 13, Figure 14 and Figure 15.

As shown in Figure 13, as the temperature increases, the phase angle generally indicates a downward trend, and the overall change is significant. The experimental findings demonstrated that as the temperature rose, the viscosity of asphalt mastic decreased while the elasticity component decreased [16]. At the same temperature, the phase angle of basalt fiber asphalt mastic was smaller than that of asphalt mastic without fiber. Compared to the asphalt mastic, the phase angle of the fiber asphalt mastic dropped by 4.8° and 3.2°, respectively, after adding 16 µm and 25 µm basalt fiber, according to the phase angle data at 76 °C. Because the fibers are covered with one another to shape a “bracket” and assumed to be part of a “network” in the asphalt mastic [38,39], a structural asphalt interfacial layer with a strong adhesion force formed on the fiber’s surface after the asphalt made contact with it, improving the bonding performance of asphalt while also limiting the mastic’s ability to deform around the fiber and lowering its fluidity [20]. Larger specific surface area fibers can absorb more asphalt and exhibit greater elastic properties. At the same weight content, 16 μm basalt fiber had more monofilaments and specific surface area than 25 μm, resulting in improved elastic characteristics in asphalt mastic, which is in accordance with the findings in reference [20].

Complex modulus test results are shown in Figure 14. As the temperature rose, the complex modulus decreased [20]. This was mainly due to the deformation and thermal effect of asphalt molecules under loading, and the motility of asphalt molecules was enhanced. Deformation was more likely to occur, which was manifested by the decrease in modulus. Results showed that its resistance to deformation when subjected to traffic loads and temperature changes was constantly weakened. Asphalt mastic with two fibers of different diameters had a larger complex modulus than that of the blank asphalt mastic at the same temperature. Following the addition of 16 µm and 25 µm basalt fibers, the complex modulus of mastic increased by 49.15% and 23.73%, respectively, when the testing temperature reached 76 °C. These outcomes demonstrated that fibers might be added to the SBS-modified asphalt to increase its deformation resistance further [32]. The complex modulus of 25 µm basalt fiber asphalt mastic was smaller than that of 16 µm fiber, indicating that 16 µm mastic presented stronger resistance to deformation, which is consistent with the research results of the references [40,41].

As the temperature rose, the rutting factor values of all three asphalt mastics decreased, indicating that the high-temperature environment weakened its resistance to rutting (Figure 15). At the same temperature, blank asphalt mastic had a lower rutting factor than the other two asphalt mastics with different diameter fibers. Taking the rutting factor data at 76 °C as an example, after adding 16 μm and 25 μm basalt fibers, the rutting factor values of fiber asphalt mastic increased by 58.11% and 28.38%, respectively. The outcomes demonstrated that fiber addition could enhance rutting resistance at high temperatures. At the same temperature, the rutting factor of 16 μm basalt fiber asphalt mastic was greater than that of 25 μm fiber, indicating smaller fiber diameter guarantees greater resistance to rutting at high temperatures.

In conclusion, basalt fiber may be added to asphalt mastic to improve its high-temperature stability, and 16 μm basalt fiber performed better than 25 μm fiber. Because of the larger specific surface area of 16 μm basalt fiber and the higher amount of monofilament fibers under the same fiber content compared to 25 μm basalt fiber, the adsorption capacity of fibers was proportional to their surface area, so fibers with a larger specific surface area can adsorb more asphalt, and fibers with smaller diameters in asphalt mastic showed better elasticity effects. Asphalt mastic had a three-dimensional network structure made of layers of evenly spaced-out fibers [38,39]. After the asphalt and fiber made contact, a layer of asphalt with a strong adhesion force formed on the fiber’s surface, improving the bonding performance between asphalt and fiber and preventing the asphalt mastic from deforming around the fiber monofilament [20]. This decreased the fluidity of asphalt mastic and increased its deformation resistance.

### 3.3. Mid-Temperature Performance Analysis of Fiber Asphalt Mastic

**Figure 16:**
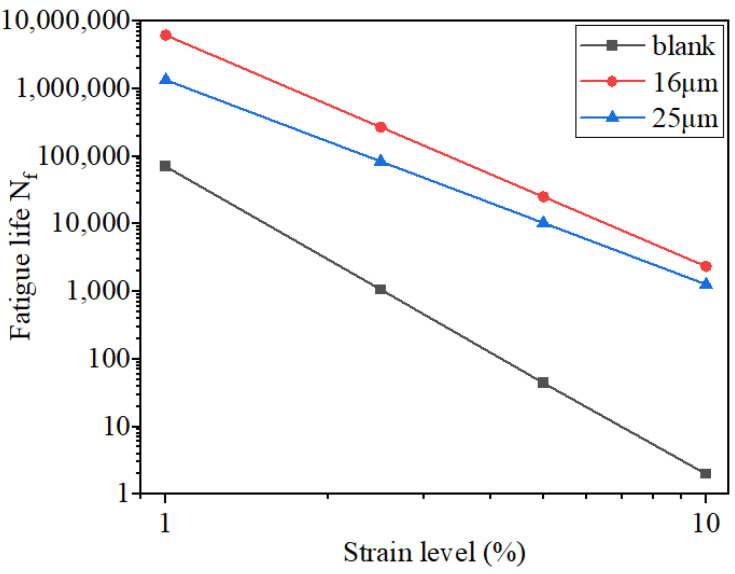
Logarithmic plot of fatigue life vs. shear strain level.

To describe the fatigue properties of asphalt, the LAS experiment was conducted using the viscoelastic continuum damage theory (VECD model). Table 7 and Figure 16 display the experimental findings.

Figure 16 shows that the fatigue resistance of asphalt mastic weakens as strain increases. The fatigue life of fiber asphalt mastic with different diameters was higher than that of the blank asphalt mastic under the same strain level. It demonstrated that fiber addition could enhance the fatigue property of SBS asphalt [19,42].

At the same strain level, 16 μm basalt fiber asphalt mastic had a higher fatigue life *N_f_* than 25 μm, indicating stronger fatigue resistance could be observed in 16 μm basalt fiber asphalt mastic. When compared to asphalt mastic with 25 μm of fiber, the fatigue life *N_f_* of asphalt mastic with 16 μm of basalt fiber rose by 220.50%, 143.62%, and 84.78% at strain levels of 2.5%, 5%, and 10%, respectively. Because under the same fiber content, the specific surface area and more monofilament of 16 μm basalt fiber could be seen than those of 25 μm basalt fiber. Therefore, 16 μm basalt fiber could absorb more asphalt to form structural asphalt [43], which slowed down the dissipation rate of the asphalt mastic to maintain good viscoelasticity for a long time and improve its fatigue life [40,41].

### 3.4. Low-Temperature Performance Analysis of Fiber Asphalt Mastic

**Figure 17:**
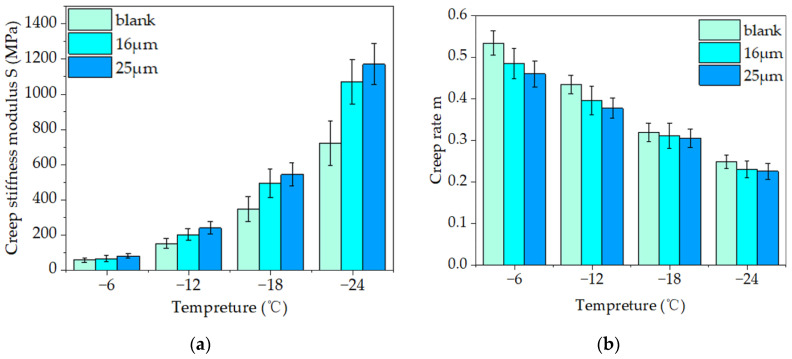
Test results of the BBR test: (**a**) creep stiffness modulus; (**b**) creep rate.

The flexural creep stiffness modulus S in the BBR test, which characterizes the flexibility of asphalt mastic, was utilized to measure the rheological property at low temperatures. With a decline in the S value, flexibility increases. The ability of the sample to relieve stress was evaluated by the creep rate m. As m increases, the stress relaxation ability of asphalt improves. Figure 17a,b display the test findings.

Figure 17a illustrates the test results for creep stiffness. As the temperature drops, the creep stiffness of the sample increases. Asphalt mastic had a lower creep stiffness modulus than basalt-reinforced mastic at the same temperature. Compared to the blank asphalt mastic, the creep stiffness values of the 16 μm and 25 μm fiber asphalt mastics increased by 32.89% and 57.24%, respectively, at −12 °C. A high creep stiffness modulus of 25 μm fiber asphalt mastic was observed at all four testing temperatures, indicating the inferior low-temperature performance of 25 μm fiber asphalt mastic compared to that of 16 μm.

As shown in Figure 17b, the creep rate of the test sample reduces with temperature drops, showing a decrease in the capacity of asphalt to release stresses at low temperatures. The creep rate of blank asphalt mastic was higher than that of the other two basalt fiber asphalt mastics at the same temperature.

In addition, the creep rate values of 16 μm and 25 μm fiber-modified asphalt mastic decreased by 9.87% and 13.13%, respectively, compared to the blank asphalt mastic at −12 °C. The findings indicated that fibers hindered the capacity of asphalt mastic to relax under stress at low temperatures [19,20]. The creep rate of 25 μm fiber-modified asphalt mastic was lower than that of 16 μm fiber-modified asphalt mastic and blank asphalt mastic at the same temperature, indicating that its low-temperature stress relaxation capacity had significantly deteriorated.

Fibers increased the creep stiffness and decreased the creep rate of asphalt mastic in the BBR test, demonstrating that fibers reduced their low-temperature rheological qualities [16]. However, the evaluation of the modified agent and fiber-modified asphalt in the BBR test was inaccurate because the test specification did not account for the material’s inherent strength [44]. Analytical methodologies should also be pursued from alternative perspectives.

### 3.5. High-Temperature Performance Analysis of Fiber Asphalt Mixture

**Figure 18:**
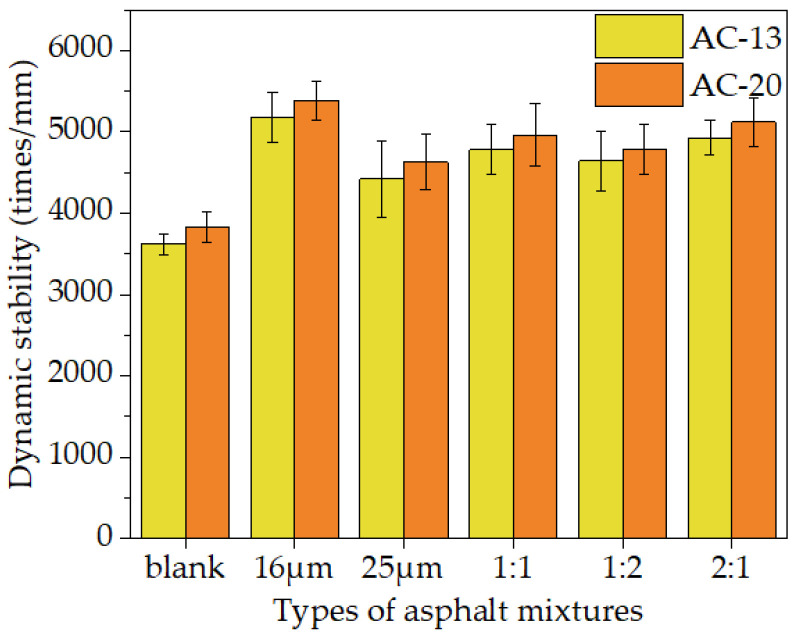
Dynamic stability results of asphalt mixtures with different fiber dosages.

The impact of fibers on the dynamic stability of mixtures is depicted in Figure 18.

It can be seen from Figure 18 that, compared to the blank mixture, asphalt mixtures with 16 μm and 25 μm basalt fiber had dynamic stability of 43.00% and 22.26% higher for the AC-13 gradation. In addition, the dynamic stability of the mixture rose by 32.30%, 28.35%, and 36.28% when the mixing scheme (16 μm: 25 μm) was 1:1, 1:2, and 2:1, respectively. The dynamic stability enhancement trend of the fiber-modified AC-20 mixture was comparable to that in the AC-13 gradation. The dynamic stability of the corresponding fiber asphalt mixture increased by 40.64%, 20.88%, 29.55%, 24.98%, and 33.62% over the blank sample. This was attributed to the fact that the viscosity of the asphalt mixture might be improved by basalt fibers’ ability to absorb some of the free asphalt and inhibit the flow ability of asphalt, as reflected in the DSR test results in Section 2.3.2. High-temperature stability can be improved by adding fibers to asphalt mastic, enhancing the properties of the asphalt mixture. The fibers in the mixture were equally dispersed and created a three-dimensional network structure simultaneously [45]. This construction could better reinforce the aggregate skeleton and strengthen the mixture. Thus, the rutting resistance performance of the mixture was improved.

Compared to asphalt mixtures with different fiber diameters, dynamic stability increased with decreasing fiber diameters. Compared to the mixtures with 25 μm basalt fiber, the dynamic stability of the AC-13 mixture with 16 μm basalt fiber rose by 16.96%. The index of the mixed-diameter fiber asphalt mixture was 6~14% bigger than that of 25 μm fiber. After fiber was used to modify the AC-20 mixture, its dynamic stability change law and improvement degree matched those of AC-13. In general, adding basalt fiber could boost dynamic stability by more than 20%. The dynamic stability values of the mixture in the remixing scheme were basically at the same level, and DS values were proportional to the content of 16 μm fiber.

### 3.6. Mid-Temperature Performance Analysis of Fiber Asphalt Mixture

**Figure 19:**
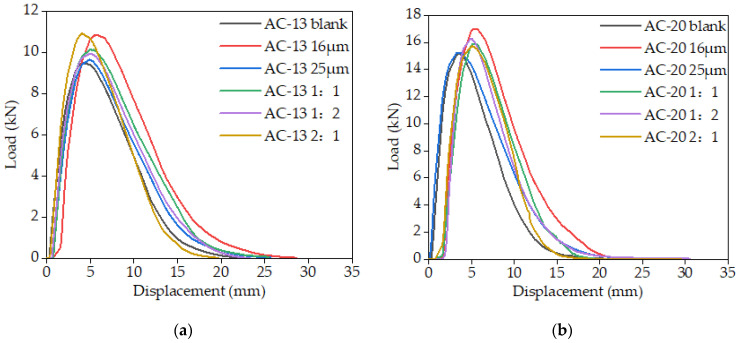
IDEAL-CT test load-displacement curve of asphalt mixture: (**a**): AC-13; (**b**): AC-20.

The results of the load-displacement curves from the IDEAL-CT test are illustrated in Figure 19.

It can be seen from Figure 19a,b that after the fiber was incorporated, the post-peak section of the curve was smoother, and the post-peak slope was also reduced. It can be explained that adding fiber could increase the toughness of the mixture, resulting in a delay in crack expansion, an extension of the cracking time of the asphalt mixture, and more energy being consumed in the cracking process to achieve the aim of enhancing crack resistance. Additionally, incorporating fiber might increase the peak load of cracking in asphalt mixtures, indicating that the fiber could strengthen the bond between aggregate and mastic and increase the energy required for crack initiation [24].

**Figure 20:**
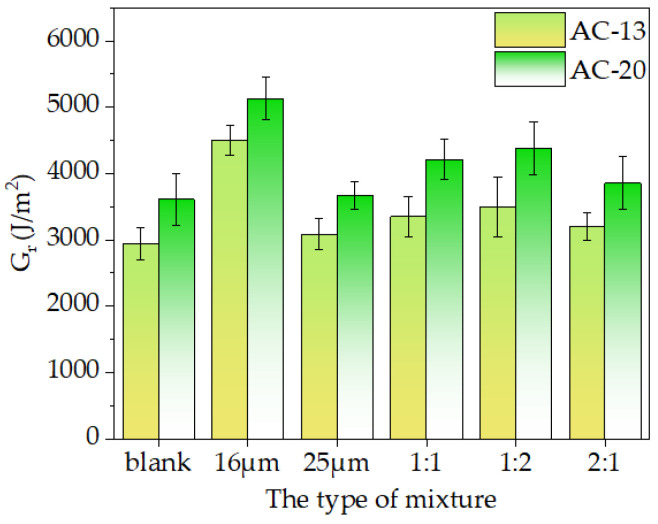
The crack initiation energy of the asphalt mixture.

**Figure 21:**
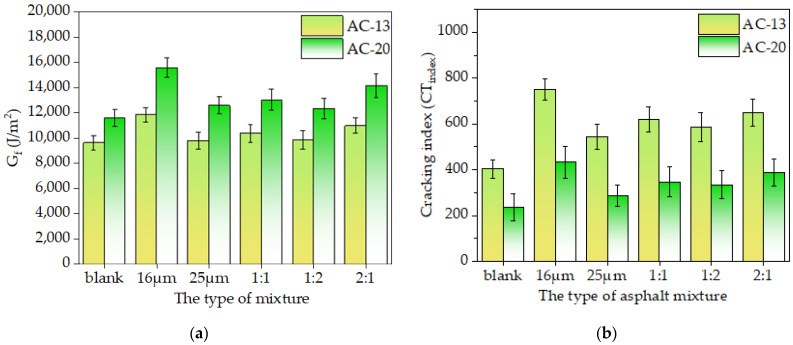
The results of the IDEAL-CT test: (**a**): Fracture energy; (**b**): Cracking index.

The load-displacement curve was processed to calculate the indicators such as *G_r_*, *G_f_*, and *CT_index_*, as shown in Figure 20 and Figure 21. The crack initiation energy *G_r_* and total fracture energy *G_f_* were used, and the crack resistance increased with *G_r_* and *G_f_* values. *CT_index_* was used to assess the resistance of asphalt mixtures to crack extension, and the higher the *CT_index_* value, the slower the crack extension and propagation, and the stronger the crack resistance achieved.

It can be seen from Figure 20 that the crack resistance of the asphalt mixture with basalt fiber was significantly higher than that of the blank asphalt mixture. The crack initiation energy (*G_r_*) of a single-doped asphalt mixture with a diameter of 16 μm or 25 μm increased by 52.97% and 4.94%, respectively, compared to the blank mixture of AC-13, while the *G_r_* values increased by 13.89%, 18.82%, and 8.61% when the compounding scheme (16 μm: 25 μm) was 1:1, 1:2, and 2:1, respectively. In addition, the rupture energy (*G_r_*) of the AC-20 followed the same change law as the AC-13 mixture. The strength and anti-crack ability of the asphalt mixture were improved by weaving a large number of basalt fibers into a three-dimensional grid structure [11]. When the mixture starts cracking, the fiber might effectively stop the crack from spreading and help the asphalt mixture withstand crack germination. This was consistent with Wu’s findings [11].

Figure 21a,b show that, as contrasted with the blank mixture of AC-13, single-doped asphalt mixtures with diameters of 16 μm or 25 μm had fracture energies *G_f_* that were 22.87% and 1.67% higher, while with the cracking indices *CT_index_* of 86.17% and 34.67% higher. The fracture energies *G_f_* of the mixture increased by 7.66%, 2.40%, and 13.94%, respectively, when the mixing scheme (16 μm: 25 μm) was 1:1, 1:2, and 2:1 asphalt mixture. The cracking index *CT_index_* values of the corresponding mixtures also increased by 53.33%, 44.94%, and 60.58%, respectively. The fracture energy *G_f_* of the AC-20 mixture followed a similar change law to that of the AC-13 mixture. Additionally, the anti-crack ability at intermediate temperatures was significantly influenced by the fiber diameter. According to the results, when fiber diameter decreased, the fracture energy (*G_f_*) and cracking index (*CT_index_*) of the asphalt mixture increased. As the AC-13 asphalt mixture with 16 μm basalt fiber was combined, the fracture energy *G_f_* and cracking index *CT_index_* rose by 20.8% and 37.4%, respectively, compared to the mixture with 25 μm fiber. However, the fracture energy *G_f_* and the *CT_index_* of the asphalt mixture mixed with two fibers of different diameters simultaneously were not much different. Still, the anti-crack ability of the asphalt mixture increased with an increase in the fiber ratio of 16 μm.

In general, the inclusion of 16 μm basalt fiber can significantly increase the anti-crack and anti-propagate abilities of the asphalt mixture [43]. However, at intermediate temperatures, the addition of 25 μm basalt fiber had minimal impact on the asphalt mixture’s resistance to cracking. The findings of the monofilament pullout test in Section 3.1 further demonstrated that at 25 °C, 16 μm basalt fiber had a higher interfacial adhesion strength than 25 μm fiber, which can further enhance the anti-crack ability of the asphalt mixtures.

### 3.7. Low-Temperature Performance Analysis of Fiber Asphalt Mixture

**Figure 22:**
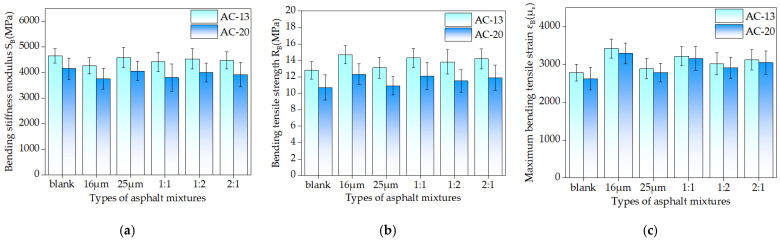
The results of low-temperature beam experiments: (**a**): Bending stiffness modulus; (**b**): Bending tensile strength; (**c**): Maximum bending tensile strain.

One of the most significant road features is the asphalt mixture’s endurance to crack at low temperatures. Asphalt mixtures are temperature-sensitive materials that are prone to cracks when they are in a low-temperature environment. In this study, a bending beam test was adopted to assess the impact of basalt fiber on the properties of the mixture at low temperatures. The results are shown in Figure 22.

As can be seen in Figure 22a–c, the addition of basalt fiber increased the bending tensile strength and maximum bending tensile strain of the mixture of the two gradations while decreasing the bending stiffness modulus. It demonstrates how the inclusion of basalt fiber could boost the flexibility of the asphalt mixture, acting as a strengthening agent and enhancing the endurance of the asphalt mixture to crack at low temperatures. The fibers were woven into the mixture and were able to adhere to the asphalt, which served to toughen the fibers. This toughening effect successfully avoided the formation of cracks, consumed some of the stress concentration, and increased the resistance of material to cracks at low temperatures [30]. Figure 22b shows that the maximum bending strain of the single-doped 16 μm or 25 μm basalt fiber asphalt mixtures was raised by 23.07% and 3.81%, respectively, in comparison to the blank AC-13 asphalt mixture. The maximum bending strain increased by 15.63%, 8.55%, and 12.25% when the asphalt mixture with a fiber ratio of 1:1, 1:2, and 2:1 was mixed. The maximum bending strain of asphalt mixtures with single-doped 16 μm and 25 μm basalt fibers increased by 25.67% and 6.37%, respectively, compared to those without fibers in the AC-20 asphalt mixtures. The maximum bending strain increased by 20.67%, 11.10%, and 16.25%, respectively, when the mixing ratio was 1:1, 1:2, and 2:1.

When fiber asphalt mixtures of various diameters were compared, it was found that the fiber diameter had a significant influence on the low-temperature crack resistance of the mixture. The smaller the fiber diameter, the lower the bending stiffness of the corresponding mixture and the higher the failure strain. This means that a smaller fiber diameter possesses better low-temperature performance. The results of the monofilament pullout test in Section 3.1 also show that under the low-temperature condition of −12 °C, the interfacial adhesion strength of 16 μm basalt fiber is 34.18% higher than that of 25 μm fiber, and 16 μm fiber could better increase the anti-crack ability of asphalt mixture [43]. When fibers of two different diameters were combined with an asphalt mixture, the properties of the corresponding mixture at low temperatures improved when the content of 16 μm fiber increased [40,41]. Generally speaking, the effect of fiber diameter on mixture cracking at low temperatures was comparable to the impact on cracking resistance at intermediate temperatures.

### 3.8. Water Stability Analysis of Fiber Asphalt Mixture

**Figure 23:**
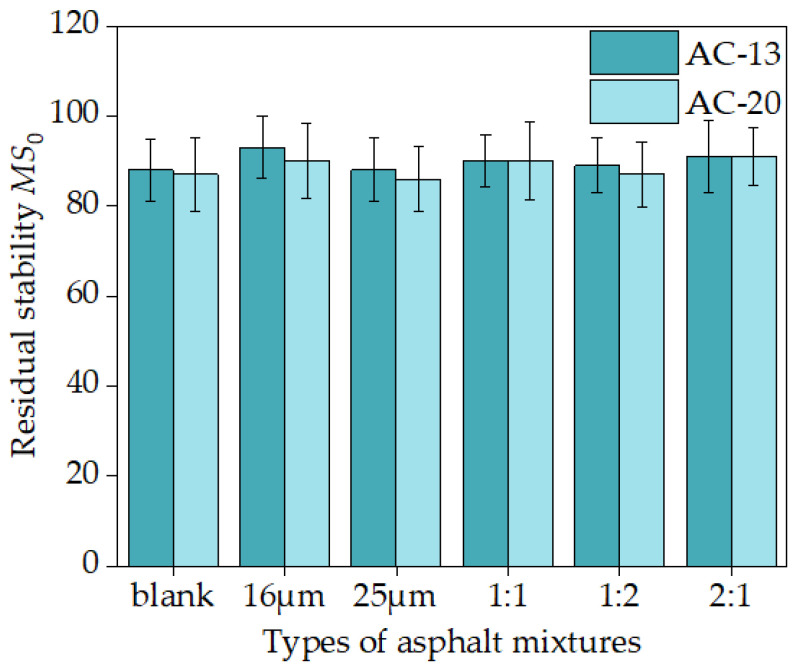
The results of the residual stability *MS*_0_ of the mixture.

It is crucial to investigate how fiber diameter affects the water stability of the mixture because the pavement is vulnerable to rain, freezing, and other types of water erosion while in use. The outcomes and standard deviations are displayed in Figure 23.

Figure 23 shows that AC-13 or AC-20 asphalt mixtures with 16 μm or 25 μm diameters had only about 4% higher residual stability *MS*_0_ in water immersion than the corresponding blank asphalt mixtures. When the remixing scheme was 1:1, 1:2, and 2:1, the residual stability of water immersion in the mixture increased by about 3%. It is clear that the water stability was not significantly affected by the fiber diameter [29].

## 4. Conclusions

In this research, two basalt fibers with diameters of 16 μm and 25 μm were chosen to make fiber asphalt mastics and asphalt mixtures (AC-13 and AC-20 grades). This study examined how well fiber sticks to the asphalt mastic and the properties of fiber-modified asphalt mastic. It also looked into how changing fiber diameter affects the stability of asphalt mixtures at high, intermediate, and low temperatures and in water baths. Within the scope of this research, the findings are as follows:

(1) The test showed that the cohesiveness between 16 and 25 μm basalt fiber and asphalt mastic was in fiber failure mode at both temperatures of −12 °C and 25 °C. At the same temperature, the interface bonding strength of 16 μm fiber with asphalt mastic is larger than that of 25 μm, which increased by 34.18% and 11.01% at −12 °C and 25 °C, respectively.
(2) Adding basalt fiber improved the SBS-modified asphalt’s rutting resistance and deformation recovery ability at high temperatures. The improvement effect of 16 mm basalt fiber was superior to that of 25 mm fiber.
(3) A linear amplitude scanning test showed that basalt fiber could improve the fatigue resistance of asphalt mastic. At strain levels of 2.5%, 5.0%, and 10%, the fatigue life *N_f_* of 16 μm basalt fiber asphalt mastic increased by 220.50%, 143.62%, and 84.78%, respectively, compared with that of 25 μm basalt fiber.
(4) According to the low-temperature bending beam rheological test, basalt fiber would lower the mastic’s low-temperature performance, which conflicts with the low-temperature performance of the mixtures, indicating the bending beam rheological test may not be suitable for fiber asphalt mastics.
(5) Adding fiber may raise the rutting stability of the asphalt mixture by 20.88% to 43.00%. As the fiber diameter shrank, the high-temperature stability improved.
(6) The addition of 16 μm basalt fiber significantly increased the resistance of the mixture to crack initiation and propagation (18~85%), whereas the addition of a single doped 25 μm basalt fiber did not considerably increase the crack resistance at intermediate-temperature and low-temperature (about 5%). The diameter of the fiber asphalt mixture was also compounded, and the more 16 μm fiber there was in the mixture, the better resistance to cracking was achieved.
(7) The water stability of the basalt fiber mixture could meet the specifications, and the fiber diameter had no appreciable impact on the water stability.

## Figures and Tables

**Table 1:** Performance indicators and test results of SBS-modified asphalt.

Properties		Specification	Test Results	Test Method [31]
Penetration (25 °C)/0.1 mm		40~70	56	T0604
Penetration index PI		−0.4~1.0	0.6	T0604
Softening point/°C		≮80	84.7	T0606
Ductility (5 cm/min, 5 °C)/cm		≮30	46	T0605
Segregation (softening spreads)/°C		≯2.5	1.5	T0661
Resilient recovery (25 °C)/%		≮65	77	T0662
Residue after RTFOT	Quality changes/%	±1.0	−0.09	T0610
	Penetration ratio/%	≮60	88	T0604
	5 °C residual ductility/cm	≮20	39	T0605

**Table 2:** Performance indices and test results of basalt fiber.

Properties	Test Results	
	16 μm	25 μm
Breaking force/cN	32.69	38.61
Elongation/%	4.68	5.06
Modulus/GPa	88	79
Density/g·cm^−3^	2.72	2.66
Water content/%	0.1	0.1

**Table 3:** Technical indicators of limestone ore powder.

Properties	Specification	Test Results	Test Method [33]
Water content/%	≤1.0	0.43	Drying method
Relative density	≥2.50	2.708	T0352
Hydrophilic coefficient	<1	0.66	T0353
Particle size range: <0.6 mm	100	100	T0351
Particle size range: <0.15 mm	90~100	93.4	T0351
Particle size range: <0.075 mm	75~100	81.6	T0351

**Table 4:** Test results of aggregate density.

Type		Apparent Relative Density (g/cm^3^)	Relative Density of Gross Volume (g/cm^3^)	Asphalt Mixture
Limestone	1#	2.785	2.731	AC-13
	2#	2.781	2.722	
Basalt	3#	2.900	2.850	
	4#	2.861	2.796	
Limestone	1#	2.718	2.686	AC-20
	2#	2.779	2.731	
	3#	2.776	2.719	
Basalt	4#	2.855	2.791	

**Table 5:** AC-13 gradation.

Screen Aggregate	Percentage of Passing through a Square Hole Sieve (mm)/%									
	16	13.2	9.5	4.75	2.36	1.18	0.6	0.3	0.15	0.075
1#	100	91.1	33.5	0.2	0.2	0.2	0.2	0.2	0.2	0.2
2#	100	98.3	83.5	4	0.1	0.1	0.1	0.1	0.1	0.1
3#	100	100	100	92.9	6.2	2.3	2	1.9	1.8	1.6
4#	100	100	100	97.7	66.9	35.5	21.1	9.9	5.5	3.9

**Table 6:** AC-20 gradation.

Screen Aggregate	Percentage of Passing through a Square Hole Sieve (mm)/%											
	26.5	19	16	13.2	9.5	4.75	2.36	1.18	0.6	0.3	0.15	0.075
1#	100	31.9	12.8	1.9	0.5	0.5	0.5	0.4	0.4	0.3	0.2	0.1
2#	100	100	100	91.1	33.5	0.2	0.2	0.2	0.2	0.2	0.2	0.2
3#	100	100	100	98.3	83.5	4	0.1	0.1	0.1	0.1	0.1	0.1
4#	100	100	100	100	100	97.7	66.9	35.5	21.1	9.9	5.5	3.9

**Table 7:** The result of the LAS test.

Specimen	Model A	Model B	*α*	2.5%*N_f_*	5%*N_f_*	10%*N_f_*
SBS	70,400	4.57	2.28	1067.157	44.323	1.985
SBS-16 μm	6,154,000	3.42	1.71	268,315.212	25,089.889	2346.64
SBS-25 μm	1,336,000	3.02	1.51	83,716.875	10,298.677	1269.985

## Data Availability

Not applicable.
